# Multimodal Fusion Image Stabilization Algorithm for Bio-Inspired Flapping-Wing Aircraft

**DOI:** 10.3390/biomimetics10070448

**Published:** 2025-07-07

**Authors:** Zhikai Wang, Sen Wang, Yiwen Hu, Yangfan Zhou, Na Li, Xiaofeng Zhang

**Affiliations:** 1 College of Information Engineering, Henan University of Science and Technology, Luoyang 471023, China; zhikaiwang@haust.edu.cn (Z.W.); wsen34155@gmail.com (S.W.); 2 Henan Key Laboratory of Robot and Intelligent System, Henan University of Science and Technology, Luoyang 471023, China; 3 National Key Laboratory of Digital and Agile Aircraft Design, Chengdu 610091, China; 4 AVIC Chengdu Aircraft Design Research Institute, Chengdu 610091, China; 5 Suzhou Institute of Nano-Tech and Nano-Bionics (SINANO), Chinese Academy of Sciences, Suzhou 215123, China; yfzhou2020@sinano.ac.cn; 6 School of Electrical Engineering and Automation, Harbin Institute of Technology, Harbin 150001, China; zxfeng@stu.hit.edu.cn

**Keywords:** biomimetic aircraft, cross-attention mechanism, optical flow, SEA-RAFT, image enhancement

## Abstract

This paper presents FWStab, a specialized video stabilization dataset tailored for flapping-wing platforms. The dataset encompasses five typical flight scenarios, featuring 48 video clips with intense dynamic jitter. The corresponding Inertial Measurement Unit (IMU) sensor data are synchronously collected, which jointly provide reliable support for multimodal modeling. Based on this, to address the issue of poor image acquisition quality due to severe vibrations in aerial vehicles, this paper proposes a multi-modal signal fusion video stabilization framework. This framework effectively integrates image features and inertial sensor features to predict smooth and stable camera poses. During the video stabilization process, the true camera motion originally estimated based on sensors is warped to the smooth trajectory predicted by the network, thereby optimizing the inter-frame stability. This approach maintains the global rigidity of scene motion, avoids visual artifacts caused by traditional dense optical flow-based spatiotemporal warping, and rectifies rolling shutter-induced distortions. Furthermore, the network is trained in an unsupervised manner by leveraging a joint loss function that integrates camera pose smoothness and optical flow residuals. When coupled with a multi-stage training strategy, this framework demonstrates remarkable stabilization adaptability across a wide range of scenarios. The entire framework employs Long Short-Term Memory (LSTM) to model the temporal characteristics of camera trajectories, enabling high-precision prediction of smooth trajectories.

## 1. Related Work

Small flapping-wing drones equipped with visual sensors offer unique advantages for executing tasks in complex environments. Due to their compact structure, high maneuverability, prominent biomimetic features, and stealth capabilities, they have garnered significant attention in recent years for applications such as low-altitude exploration, post-disaster inspection, and environmental monitoring. However, compared to fixed-wing and rotary-wing drones, flapping-wing drones encounter frequent vibrations and complex disturbances stemming from the periodic flapping of their wings during flight. These factors give rise to severe jitter, local blurring, and structural distortion in the video sequences captured by onboard cameras. Such issues significantly undermine the accuracy and robustness of subsequent perception and analysis tasks. Therefore, designing a robust video stabilization algorithm that adapts to the motion characteristics of flapping-wing platforms is of great importance for enhancing the practicality and effectiveness of their visual systems in task execution.

Current research on video stabilization algorithms predominantly relies on publicly available datasets. Notable examples of influential and representative benchmarks include NUS [1], DeepStab [2], MotionStab [3], Selfie [4], and DeepFVS [5]. However, these existing public datasets exhibit significant limitations in application scenarios, sensor configurations, and motion diversity. For instance, the Selfie dataset is tailored for video stabilization in selfie scenarios. The NUS dataset [1], despite encompassing diverse scenes—including simple motions, rapid rotations, zooming, large parallax, driving, crowds, and running—lacks synchronized IMU data, rendering it inapplicable to visual-inertial fusion stabilization algorithms. The DeepStab [2] and MotionStab [3] datasets provide stable-unstable video pairs, yet these are confined to artificially induced shaky scenes captured with handheld mechanical stabilizers. Consequently, these datasets feature monotonous motion patterns and lack inertial data from real-world complex motion environments. Although the DeepFVS [5] dataset offers IMU sensor data, its recording scenarios are confined to handheld camera footage in daily-life settings, thus failing to capture the intricate motion dynamics characteristic of real-world flight conditions.

In terms of video stabilization algorithms, significant progress has been made in both online and offline video stabilization methods in recent years. Most techniques are designed to remove or mitigate undesired high-frequency jitter by estimating the camera trajectory from 2D, 2.5D, or 3D perspectives and synthesizing smooth camera trajectories. Early 2D methods tracked features across several frames and then smoothed these feature trajectories to stabilize the video [6,7]. However, during significant camera motions, features often vanish from the image as the camera moves, rendering it challenging to obtain long feature trajectories [8]. Consequently, these methods are only applicable to fixed-view scenarios with jitter, proving inadequate for situations involving substantial scene changes, such as when using handheld devices during walking or running. Some methods compute motion models, such as affine [9] or homography [10,11], between adjacent frames, relaxing the requirement from relying on long feature trajectories to merely matching features between consecutive frames [12]. Subsequently, the motion across all image frames is aggregated into a 2D camera motion trajectory, providing a robust alternative to feature-trajectory-based methods and significantly enhancing algorithmic robustness. For inter-frame motion modeling, methods such as hybrid homography [13], mesh-based [1,7,14], and optical flow [15,16] models have been proposed to handle scenes with significant depth variations. Additionally, specialized methods have been developed to address specific stabilization tasks, including selfie videos [4], 360-degree videos [17], and hyperlapse videos [18].

Furthermore, 3D-based methods rely on 3D camera motion or scene structure to achieve stabilization. The 3D structure can be computed from videos using Structure from Motion (SfM) [19] or obtained from auxiliary hardware like depth cameras [20], gyroscope sensors [21], or light field cameras [8]. However, fully 3D-based stabilization methods exhibit lower reliability and incur prohibitively high computational costs [19]. To address this issue, some 3D methods incorporate 3D constraints, such as subspace projection [22] and epipolar geometry [23], to reduce computational load and relax the requirement for full 3D reconstruction [24]. These approaches are commonly referred to as 2.5D methods. Generally, 3D-based methods can better handle scene parallax by accurately reconstructing the camera’s real-world motion, provided that the underlying 3D structure is precisely recovered. However, the aforementioned video stabilization methods are predominantly designed, trained, and evaluated on datasets from handheld cameras in daily-life scenarios. As a result, their performance is suboptimal when applied to videos captured under the intense vibrations of flapping-wing drones. To tackle the stabilization challenges in such demanding conditions, Ye et al. [25] proposed a periodic-jitter-aware digital video stabilization method. This approach adaptively adjusts the consistency of jitter frequencies using historical trajectories and employs a smooth sampling-interpolation averaging strategy for stabilization.

With the development of artificial intelligence technology, learning-based methods have been widely applied in image and video stabilization. Deep learning-based methods take video frames as input and directly output stabilized frames. These methods are typically trained on specialized datasets that contain pairs of unstable and stable frames. Deep learning-based motion models have demonstrated remarkable performance in motion estimation. Prominent examples include deep learning methods for homography estimation [26,27,28] and their extensions to dense motion estimation based on deep meshes [29,30,31]. Compared to traditional solutions, these approaches maintain effectiveness in low-texture and low-light scenarios, demonstrating robust performance. Wang et al. [2] proposed an end-to-end learning framework for video stabilization. This framework optimizes the network by leveraging stability and temporal loss terms. Additionally, the authors curated the first dataset consisting of pairs of unstable and stabilized video sequences. Xu et al. [32] employed an adversarial network to generate target images, which guide the warping process without explicitly estimating or smoothing the camera path. Yu et al. [33] derived flow fields from initial optical flow estimates to achieve precise per-pixel motion compensation. Zhao [34] proposed PWStableNet, which employs a cascaded multi-level encoder-decoder architecture to compute per-pixel warping maps for video stabilization. However, deep learning methods encounter generalization challenges due to insufficient data diversity; for example, the DeepStab dataset consists of merely 60 videos. Yu et al. [35] substituted traditional optimizers with CNNs that are designed to learn and adapt to individual input examples, rather than overfitting them. Zhao et al. [36] proposed an iterative optimization-based approach, integrating a full-frame outpainting network with a multi-frame fusion strategy to achieve full-frame stabilization. Peng et al. [37] presented RStab, a 3D multi-frame fusion framework that leverages volume rendering to generate full-frame stabilized images while preserving scene structure.

Current learning-based methods predominantly stabilize videos by extracting features from video content and optical flow. However, their performance heavily relies on training datasets and tends to induce video distortion during significant foreground motion of aircraft. Furthermore, while incorporating hardware such as gimbals could effectively mitigate image jitter, the limited payload capacity of flapping-wing UAVs presents a significant challenge for mounting hardware stabilization platforms. Consequently, this paper proposes a software-based stabilization approach. We introduce a multimodal fusion image stabilization algorithm for flapping-wing UAVs that utilizes a gyroscope to compensate for camera motion and employs optical flow to correct residual motion-induced distortions in scene geometry. To address the issue of poor image acquisition quality caused by the severe jitter characteristic of flapping-wing UAVs, a multimodal signal fusion video stabilization framework is proposed. This framework effectively integrates image features with inertial sensor features to predict smooth and stable camera poses. To address this, this paper proposes a multi-modal fusion stabilization algorithm for flapping-wing aircraft, which utilizes gyroscope data to compensate for camera motion and employs optical flow to rectify residual motion in scene geometry.

## 2. Construction of the Flapping-Wing Drone Video Stabilization Dataset

Bio-inspired flapping-wing drones, as a novel class of aerial vehicles, exhibit highly dynamic and rapidly varying attitudes during flight, accompanied by complex and diverse flight maneuvers. Consequently, the video data captured by their onboard visual systems is highly susceptible to severe jitter interference. To address the inadequacy of existing datasets in effectively supporting stabilization algorithm research for this novel application scenario, a dedicated visual-inertial fusion stabilization dataset based on bio-inspired flapping-wing drones has been constructed. This dataset is acquired by equipping flapping-wing drones with high-frame-rate visual sensors and high-precision six-axis IMUs, enabling the synchronous collection of video and inertial data under real-world flight conditions. It comprehensively captures the unique motion characteristics and inertial disturbances inherent to flapping-wing flight dynamics. This dataset not only enriches the data sources for stabilization algorithm research but also provides a valuable experimental foundation and validation environment for advancing visual-inertial fusion stabilization technologies in the domain of unmanned aerial vehicles.

### 2.1. Dataset Acquisition Platform and Hardware Setup

**Figure 1:**
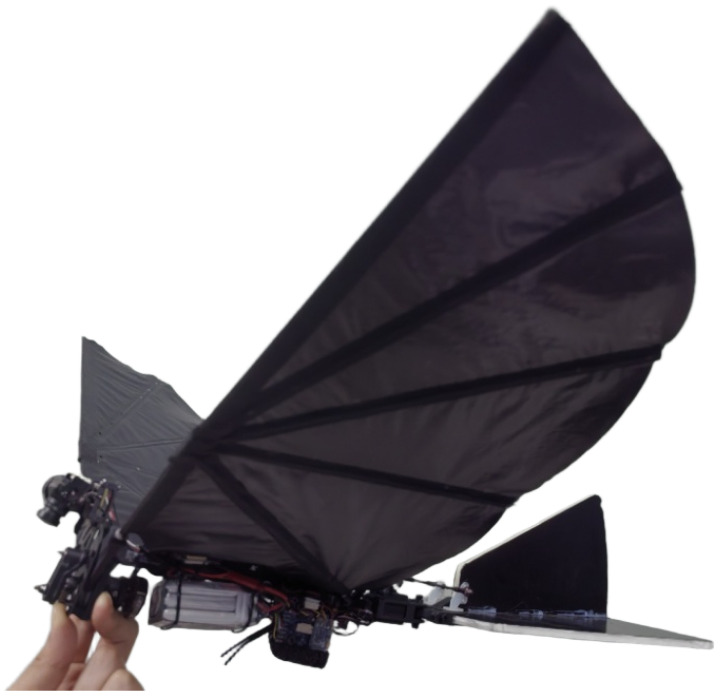
Biomimetic flapping wing aircraft data acquisition platform.

To construct a video stabilization dataset that captures realistic and complex flight dynamics, this study developed a dedicated visual-inertial data acquisition platform based on bio-inspired flapping-wing drones, as shown in Figure 1. The selected flapping-wing drone features a wingspan of approximately 600 mm, a fuselage length of 520 mm, a maximum flight speed of 15 m/s, and an endurance of up to 20 min. These specifications render it well-suited for video data acquisition across diverse and complex flight scenarios.

The selected visual sensor is the high-performance FPV camera, Avatar Pro Camera, from Walksnail. Its specific parameters are shown in Table 1.

Simultaneously, the visual sensor is integrated with a high-precision six-axis IMU, which records time-stamped IMU data in real-time during flight and simultaneously generates log files. By integrating the bio-inspired flapping-wing drone with the aforementioned visual and IMU sensors, this hardware platform effectively ensures real-time synchronization between video and IMU data, providing high-precision, high-frequency inertial and visual data for robust analysis.

### 2.2. Data Acquisition Pipeline and Preprocessing

To ensure that the collected data comprehensively captures the complex motion characteristics of real-world flight environments, a typical urban outdoor environment was selected for the video stabilization data collection of the bio-inspired flapping-wing drone. Data collection was conducted under diverse lighting conditions, encompassing bright sunny days and overcast, low-light scenarios, to evaluate the generalization capabilities of visual stabilization algorithms across varying illumination levels. Additionally, flights were executed in different wind conditions, including tailwinds and headwinds, to assess the algorithms’ adaptability to different degrees of jitter induced by varying aerodynamic forces. The flight altitude of the flapping-wing drone was maintained within the range of 30 to 80 m to simulate typical operational heights in real-world applications.

During the data acquisition process, the flapping-wing drone executed a diverse array of complex flight maneuvers, including rapid circling, intense jittering, sharp turns, roll and pitch variations, to capture realistic and comprehensive video jitter characteristics. A total of 48 video clips with synchronized IMU sensor logs were collected, covering diverse environments, including densely built urban areas, open terrains, and clear skies. This diverse environmental selection effectively enhances the applicability and robustness of video stabilization algorithms in real-world scenarios.

To facilitate the development and testing of visual-inertial fusion stabilization algorithms, the collected video and raw IMU data underwent necessary synchronization and preprocessing. First, during the data acquisition process, the video and IMU data were synchronously recorded using a unified high-precision timestamp. The precise timestamp information in the IMU log was then employed to achieve strict alignment between the visual and inertial data, ensuring the temporal consistency of visual frames with the corresponding IMU sensor data. Second, the raw IMU data was converted into physically meaningful units using the scaling factors specified in the IMU log (gyroscope scaling factor: 0.00053263221; accelerometer scaling factor: 0.00012207031). The gyroscope data was transformed to radians per second (rad/s), while the accelerometer data was converted to meters per second squared (m/$s^{2}$), enabling a more intuitive depiction of the attitude changes and motion characteristics during flight. Additionally, the original resolution and frame rate of the collected videos were preserved without any cropping or compression, thereby ensuring the authenticity and integrity of the visual information.

### 2.3. Dataset Composition

**Figure 2:**
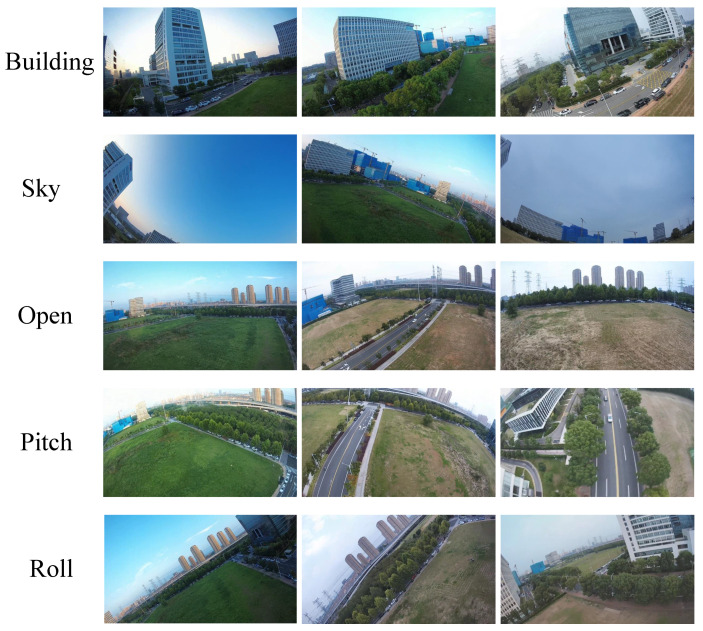
Stable dataset of flapping wing videos.

The self-constructed video stabilization dataset comprises 48 high-definition video clips, each with a uniform resolution of 1080 p (1920 × 1080 pixels) and a frame rate of 60 frames per second (fps). The duration of each video clip ranges from 10 to 20 s. Sample frames from the dataset are shown in Figure 2. Figure 2 presents several examples from the five scene categories in the FWStab Dataset: architecture, sky, open space, pan, and roll. The dataset synchronously provides high-frequency six-axis inertial measurement data recorded by the visual sensor’s built-in IMU, with a sampling frequency of 500 Hz. Notably, the FWStab Dataset employs the Walksnail Avatar Pro Camera—specifically selected for its lack of an Optical Image Stabilization (OIS) module—for capturing footage. This deliberate choice prevents interference from OIS systems with the original motion information, ensuring the collected data is more authentic and representative.

All videos in the dataset are raw, unstabilized footage captured by the flapping-wing drone during flights in diverse environments, including densely built urban areas, open terrains, and clear skies. Without any post-stabilization processing, these videos authentically and comprehensively reflect the intense jitter characteristics generated by the drone’s dynamic flight maneuvers and attitude variations. Additionally, the dataset provides high-frequency six-axis inertial measurements recorded by the IMU embedded in the visual sensor, with a sampling rate of 500 Hz. The IMU data comprises triaxial angular velocity from the gyroscope $\left(g_{x},g_{y},g_{z}\right)$ and triaxial linear acceleration from the accelerometer $\left(a_{x},a_{y},a_{z}\right)$ during flight. To ensure the authenticity and fidelity of the collected data, the Walksnail Avatar Pro Camera, specifically one without an Optical Image Stabilization (OIS) module, was deliberately selected for data acquisition. This selection effectively prevents the OIS system from distorting or altering the raw motion dynamics, ensuring that the captured data accurately represents the drone’s inherent motion characteristics. In comparison to the DeepFVS dataset, which employs OIS-equipped smartphones, this dataset exhibits higher rawness and a more genuine portrayal of motion, making it a more reliable benchmark for the development of stabilization algorithms.

## 3. Deep-Learning-Based Multimodal Signal Fusion Video Stabilization Networ

### 3.1. Network Architecture Design

**Figure 3:**
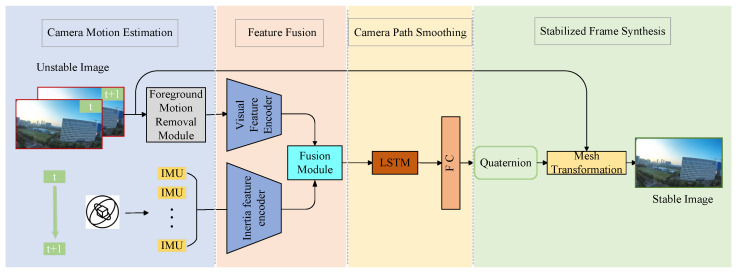
The overall framework of multi modal signal fusion video stabilization.

The proposed method in this paper, as illustrated in Figure 3, divides the video stabilization task into four main stages. In the motion estimation phase, inertial data $\left(g_{x},g_{y},g_{z},a_{x},a_{y},a_{z}\right)$ representing camera motion is extracted from the IMU and preprocessed. Simultaneously, optical flow motion between adjacent video frames is calculated to derive the camera’s rotational trajectory from the image sequence. Subsequently, optical flow motion caused by foreground dynamic objects is filtered out to minimize interference with the global motion. In the feature fusion stage, the optical flow is encoded using 2D convolutional layers to extract image motion features. These features are then fused with the features derived from the IMU data, which are encoded by an LSTM. In the camera path smoothing stage, the feature fusion module outputs fused features that integrate image and inertial sensor data. These features are then fed into an LSTM to predict smooth camera rotations (relative quaternions). Finally, the quaternions are utilized to transform the unstable image sequence into a stable one through grid transformation, and a stabilized video is synthesized.

### 3.2. Multimodal Signal Fusion Module

**Figure 4:**
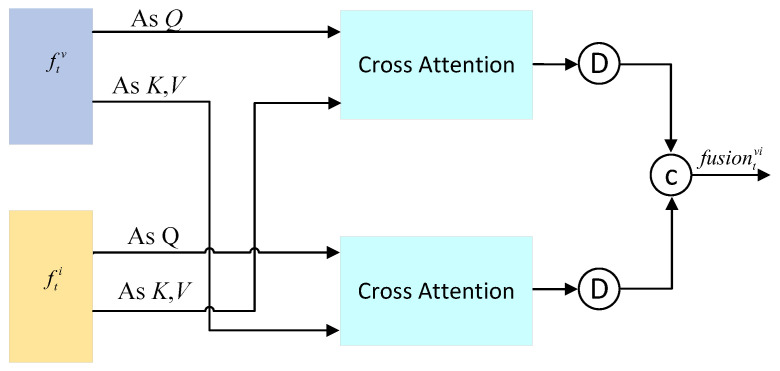
Multi modal signal fusion module.

To integrate image and inertial features, we propose a dual-branch cross-attention multimodal fusion framework, as shown in Figure 4. This framework is specifically designed to fuse optical flow features with IMU features, thereby enhancing the accuracy of camera rotation estimation in video stabilization tasks. This framework capitalizes on the complementary nature of optical flow and IMU data by constructing separate optical flow-driven and IMU-driven branches. It employs the Cross-Attention Mechanism to enable feature interaction and fusion, thus facilitating the full utilization of information from different modalities.

In the feature extraction stage, 2D convolutional layers are used to extract optical flow features from which the interference of dynamic foreground motion has been removed, yielding a feature representation that captures the global motion (camera rotation) of the images. To mitigate noise in the IMU data, an LSTM network is employed to extract the camera’s rotational features from the IMU signals.

First, the optical flow information is encoded. Let the optical flow field be denoted as $OF\in R^{H\times W\times 2}$, where H and W denote the image resolution (height and width), and the channel dimension of 2 corresponds to the (u,v) directional components of the optical flow. A 2D convolutional network is then applied to encode this optical flow field, extracting discriminative motion features:

(1) $$f_{t}^{v}=CNN\left(OF\right)\in R^{T\times d}$$

Here, $f_{t}^{v}$ denotes the extracted optical flow features, where *T* represents the number of temporal steps and *d* is the dimensionality of the embedded feature space. The IMU data, consisting of measurements from the gyroscope and accelerometer, has its raw sensor data represented as:

(2) $$S_{IMU}=\left[\omega_{x},\omega_{y},\omega_{z},a_{x},a_{y},a_{z}\right]\in R^{T\times d}$$

Temporal features are extracted from the IMU data using an LSTM network:

(3) $$f_{t}^{i}=LSTM\left(S_{IMU}\right)\in R^{T\times d}$$

In the two branches, the optical flow features (the IMU features) $f_{t}^{v(i)}$ are taken as queries (Query, Q), and the IMU features (the optical flow features) $f_{t}^{i(v)}$ are taken as key-value pairs (Key, Value, K, V), with the cross-attention mechanism employed:

(4) $$Attention\left(Q,K,V\right)=soft\max\left(\frac{QK^{T}}{\sqrt{d}}\right)V$$

Feature fusion is performed to obtain the fused optical-flow-enhanced features (IMU-enhanced features):

(5) $$f_{t}^{vi(iv)}=Attention\left(f_{t}^{v(i)},f_{t}^{i(v)},f_{t}^{i(v)}\right)$$

The enhanced features $f_{t}^{vi}$ and $f_{t}^{iv}$ from the two branches are downsampled and then concatenated to obtain the fused features.

(6) $$fusion_{t}^{vi}=concat\left(Down\left(f_{t}^{vi}\right),Down\left(f_{t}^{iv}\right)\right)$$

Finally, the fused features are fed into an LSTM to predict the stabilized camera rotation trajectory.

The flapping-wing motion, realized through periodic upstroke-downstroke cycles, wing pitching, and fanning, directly manifests in the acceleration and angular velocity signals of the IMU sensor. Specifically, the vertical vibration generated by wing flapping induces periodic acceleration variations along the Z-axis, accompanied by angular velocity changes around the X and Y axes. For the video stabilization algorithm, accurate parsing of these IMU signals helps identify and compensate for high-frequency jitters caused by flapping, thereby improving the precision of motion estimation and the effectiveness of image stabilization.

### 3.3. Foreground Motion Removal Module

To accurately and effectively eliminate motion artifacts caused by dynamic foreground objects in optical flow, a foreground motion removal module has been designed. This module is crucial for subsequent refinement of the optical flow field for downstream stabilization tasks.

A common solution for detecting distinct motions is to employ motion segmentation methods [14]. However, motion segmentation itself is a complex computer vision task. Many approaches rely on long feature trajectories extracted from multiple frames, which can be computationally intensive. Although two-frame-based motion segmentation methods [13,38] exist, they often struggle to handle large foreground objects due to insufficient motion contrast between adjacent frames.

**Figure 5:**
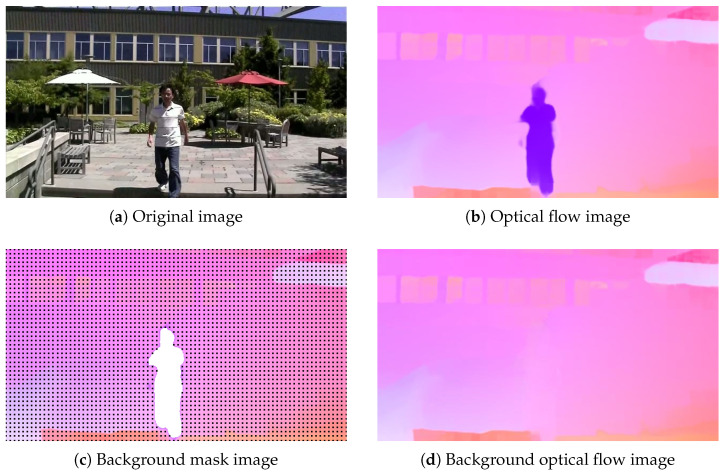
Remove foreground motion module.

The objective of this module is to mitigate the impact of dynamic objects on camera motion estimation. Motion induced by camera jitter is spatially consistent, whereas motion caused by dynamic objects is spatially inconsistent. As illustrated in Figure 5b, the optical flow map reveals distinct motion patterns between foreground dynamic objects and background motion. Consequently, when the image contains foreground motion that differs from the background motion, dynamic foreground objects can interfere with subsequent global motion estimation, thereby degrading the network’s accuracy in estimating global motion. To address this, a classification-based approach is employed to isolate foreground motion, retaining only the global motion information relevant to camera motion estimation.

Let the frames of the input video be denoted as $\left\{f_{i}|\forall i\in \left[1,N\right]\right\}$, where $f_{i}$ represents the i-th frame of the video and *N* is the total number of frames. First, the SEA-RAFT [39] algorithm is employed to compute the optical flow between consecutive frames.

(7) $$OF_{i}=SEARAFT\left(f_{i},f_{i+1}\right)$$

SEA-RAFT [39] is a simplified variant of RAFT [40], offering faster runtime and robust performance. Previous stabilization methods [7,34] relied on heuristic rules to mitigate noise induced by dynamic objects, for example, by excluding motion from dynamic objects via predefined thresholds. However, these approaches require manual tuning of thresholds for each unstable video, as fixed thresholds fail to generalize across diverse jittery scenarios. Additionally, most methods [32,41] rely on the number and quality of extracted feature points. However, videos captured by flapping-wing drones often contain large textureless areas like the sky and experience severe jitter, leading to issues such as loss of feature points and uneven distribution. This results in a scarcity of feature points and poor quality, making it challenging to estimate accurate camera motion. Therefore, the foreground motion removal module uses the KMeans algorithm to classify the foreground motion and background motion in the optical flow into two categories, namely

(8) $$c_{i}^{k}=\left\{KMeans\left(OF_{i}\right),\forall i\in \left[1,N\right],k\in 0,1\right\}$$

Let $c_{i}^{k}$ denote the result of using KMeans to cluster the optical flow $OF_{i}$ between the i-th and (i+1)-th frames into two clusters,

(9) $$I_{i}^{k}=\left\{\left(h_{ij},w_{ij}\right):c_{i}^{k},1\leq h_{ij}\leq H_{i},1\leq w_{ij}\leq W_{i},k\in 0,1\right\}$$

where $I_{i}^{k}$ represents the set of height and width indices of all points from the two clusters, $h_{ij}$ and $w_{ij}$ denote the height and width indices of the i-th optical flow respectively, and $H_{i}$ and $W_{i}$ represent the height and width values of the image respectively.

To accurately estimate camera motion and obtain a smooth global motion representation, processing the foreground motion in the optical flow is essential. Directly applying Gaussian smoothing to the foreground motion contours in the optical flow would propagate the motion of dynamic foreground objects to the background, degrading the accuracy of global motion estimation. Therefore, a mask $M_{i}^{0}=I_{i}^{0}$ is utilized to extract the background motion from the optical flow, denoted as $OF_{i}^{1}=OF_{i}\left(M_{i}^{0}\right)$, while discarding discontinuous optical flow vectors. In $M_{i}^{0}$, values of 0 and 1 represent the foreground and background regions, respectively (where $M_{i}^{1}=I_{i}^{1}$ indicates that the mask of the blank area is the opposite of $M_{i}^{0}$). Figure 5 shows an example of foreground motion removal. Specifically, Figure 5a represents the input video frame (from the DeepStab public dataset). Figure 5b depicts the original optical flow. Figure 5c illustrates the separation of foreground motion using the KMeans clustering algorithm (the matrix composed of black points is the available optical flow region mask, while the white area is the outlier mask). Figure 5d shows the result of filling discontinuous optical flow motion vectors with nearby information.

(10) $$N\left(OF_{i}^{1}\right)=\frac{1}{k\times k}\sum_{i,j}F_{i}^{1}\left(i,j\right)$$

(11) $$OF_{i}^{1}\left(i,j\right)=\left\{\begin{array}{cc} N\left(OF_{i}^{1}\right)\left(i,j\right), & \mathrm{if}\;M_{i}^{0}\left(i,j\right)=1\\ OF_{i}^{1}\left(i,j\right), & \mathrm{if}\;M_{i}^{0}\left(i,j\right)=0 \end{array}\right.$$

Here, $N(OF_{i}^{1})$ represents the neighborhood average of optical flow for the inpainting points. We set k=3 as the parameter for the neighborhood calculation. When the mask $M_{i}^{0}\left(i,j\right)=1$, the original optical flow is retained. When $M_{i}^{0}\left(i,j\right)=0$, the smoothed global optical flow vector is obtained by calculating the average of the neighboring optical flow values.

### 3.4. Loss Functions

To smooth the camera trajectory and generate stable video sequences, we define the following loss functions to train the network. These loss functions can be evaluated in an unsupervised manner without requiring ground-truth stabilized video data.

(1) **Smoothness Loss**

The zero-order term constrains the instantaneous rotational changes between adjacent frames to prevent abrupt discontinuities, while the first-order term constrains the acceleration of rotational velocity to ensure second-order differentiability of the motion trajectory, thereby enhancing visual smoothness.

(12) $$L_{\mathrm{smooth}}=\lambda_{C^{0}}\sum_{t}{∥q_{t}-q_{t-1}∥}_{\mathrm{geo}}+\lambda_{C^{0}}\sum_{t}{∥\left(q_{t}⊗q_{t-1}^{-1}\right)-\left(q_{t-1}⊗q_{t-2}^{-1}\right)∥}_{2}$$

Here, the geometric distance of the quaternion is defined as:

(13) $${∥q_{a}-q_{b}∥}_{\mathrm{geo}}=\min\left({∥q_{a}-q_{b}∥}_{2},\;{∥q_{a}+q_{b}∥}_{2}\right)$$

Here, the parameters are set as $\lambda_{C^{0}}=1.0$ and $\lambda_{C^{1}}=4.0$ This setting addresses training instability issues caused by quaternion sign flipping. By applying physically inspired smoothness constraints, the method generates stable videos that align with human visual perception.

(2) **Distortion Loss**

While pursuing smoothness, it is critical to maintain consistency with real-world physical motion to prevent geometric distortions caused by over-stabilization (e.g., structural warping such as building bending). When the deviation between the virtual camera pose and true camera pose exceeds a threshold $\beta_{0}=6^{{}^{\circ}}$, a strong penalty is enforced via the steep gradient of a sigmoid function, suppressing excessive stabilization artifacts to preserve geometric fidelity.

(14) $$L_{\mathrm{distort}}=\frac{1}{1+e^{-\beta_{1}\left(\theta -\beta_{0}\right)}}\cdot {∥\theta ∥}_{2},\;\theta =\mathrm{Angle}\left(q_{v}⊗q_{\gamma }^{-1}\right)$$

Additionally, this approach corrects distortion caused by the rolling shutter effect, thereby ensuring geometric consistency in the stabilized video.

(3) **Boundary Prominence Loss**

By applying Gaussian weighting to account for boundary overlaps across future N frames, the network is compelled to predict temporally consistent cropping windows. The ReLU activation function is employed to penalize over-cropping that exceeds a threshold α, while allowing boundary adjustments within a reasonable range to preserve visual coherence.

(15) $$L_{protrude}={\sum_{i=0}^{N}\omega_{i}\cdot ReLU\left(\frac{Overlap\left(B_{t},B_{t+1}\right)-\alpha }{\alpha }\right)}^{2}$$

Here, $B_{t}$ denotes the valid bounding box after stabilizing the *t*-th frame. $\omega_{i}$ represents the Gaussian decay weight with parameters (σ = 2.5, *N* = 10) α = 0.85. This configuration reduces black borders while avoiding excessive cropping that would otherwise discard valid content. By incorporating a look-ahead constraint, the method ensures natural transitions of frame boundaries during continuous video playback, maintaining temporal coherence.

(4) **Optical Flow Consistency Loss**

By enforcing consistency between forward and backward optical flow, ambiguities in occluded regions are mitigated. A dynamic object mask $V_{t}$ is employed to exclude interference from moving object regions, suppressing artifacts caused by dynamic objects and enhancing robustness in complex scenes.

(16) $$L_{flow}=\sum_{t}\left(\frac{{∥w\left(F_{t\to t+1},M_{\nu }\right)-F_{t\to t+1}^{stab}∥}_{1}}{|V_{t}|}+EPE\left(F_{t\to t+1}^{stab},F_{t+1\to t}^{stab}\right)\right)$$

(5) **Content Reconstruction Loss**

To preserve global structure while retaining fine local details, structural similarity is computed across multiple scales of an image pyramid. Gradient difference constraints are applied to enforce edge sharpness, thereby preventing blurring artifacts during the stabilization process.

(17) $$L_{recon}=\sum_{s\in \left\{1,0.5,0.25\right\}}(SSIM(I_{t}^{raw},w(I_{t}^{stab},M_{v}^{-1},s))+\lambda_{grad}{∥\nabla I_{t}^{raw}-\nabla w_{t}∥}_{1})$$

This approach prevents over-smoothing-induced detail loss to ensure the visual quality of the stabilized video. Additionally, multi-scale processing enhances adaptability to varying motion magnitudes, ensuring robustness across diverse dynamic scenarios.

By combining the aforementioned loss functions, the total loss of the network is formulated as:

(18) $$L=\lambda_{smooth}L_{smooth}+\lambda_{distort}L_{distort}+\lambda_{protrude}L_{protrude}+\lambda_{flow}L_{flow}+\lambda_{recon}L_{recon}$$

The weight parameters of loss terms (e.g., $\lambda_{smooth}$, $\lambda_{distort}$) serve to balance the influence of different optimization objectives during training: $\lambda_{smooth}$ controls the proportion of smoothness loss, primarily suppressing abrupt trajectory fluctuations to ensure motion continuity. $\lambda_{distort}$ restricts image distortion caused by geometric transformations, preventing severe warping or stretching during stabilization. $\lambda_{flow}$ and $\lambda_{recon}$ constrain optical flow consistency and image reconstruction quality, respectively. The above parameters were all determined through empirical tuning to achieve a reasonable balance between stability and maintaining image authenticity.

(6) **Multi-Stage Training**

Simultaneously optimizing all loss terms often makes it difficult for the network to learn such nonlinear relationships, leading to convergence issues. To address this, multi-stage training is employed. In the first stage, only the smoothness loss $L_{smooth}$ is optimized using the Adam optimizer with a learning rate of 0.001. This allows the network to rapidly establish foundational motion estimation capabilities while avoiding multi-objective conflicts. In the second stage, boundary and distortion constraints (i.e., distortion loss $L_{distort}$ and boundary loss $L_{protrude}$) are introduced to refine motion trajectories. The learning rate is adjusted to 0.0005, and gradient clipping is applied. In the final stage, all loss terms are enabled, with the learning rate set to 0.0001. Each stage is trained for 200, 300, and 500 iterations, respectively.

## 4. Experimental Results and Analysis

### 4.1. Dataset

NUS Dataset [1]: The NUS video stabilization dataset consists of 174 videos with a resolution of 640×480, covering seven different scenarios: simple, fast rotation, zooming, large parallax, driving, crowded scenes, and running.

DeepStab Dataset [2]: The DeepStab Dataset comprises approximately 60 pairs of stabilized and unstabilized videos, captured using two cameras with handheld mechanical stabilizers. One camera was rigidly mounted (collecting unstabilized footage), while the other employed an active handheld stabilizer to synchronously record stabilized videos.

MotionStab Dataset [3]: This dataset encompasses five distinct scenarios: general scenes, zooming, crowded environments, time-varying scenes, and rapid rotations. A total of 110 stabilized videos were captured using a smartphone mounted on a handheld mechanical stabilizer. To generate unstable counterparts, motion transformations derived from unrelated shaky videos were applied to each frame of the stabilized videos, artificially inducing jitter. The dataset thus comprises stable-unstable motion pairs constructed from the original stabilized videos and their synthetically destabilized versions.

DeepFVS Dataset [5]: In the paper [5], the authors compiled a video stabilization dataset with gyroscope and OIS (Optical Image Stabilization) data. The dataset is split into two parts: a test set with 34 videos and their corresponding sensor data files, and a training set made up of 16 sensor data-related files.

The Self-Constructed FWStab Dataset is captured using a custom-built flapping-wing drone (as shown in Figure 1). The recording device is the Walksnail Avatar Pro Camera, which integrates an IMU sensor for synchronized motion data acquisition. Unlike the DeepFVS dataset, which relies on OIS-equipped smartphones, the FWStab dataset intentionally uses a non-OIS camera to eliminate distortions introduced by optical stabilization modules. Additionally, the IMU readings are strictly aligned with the start and end timestamps of video frames, ensuring precise spatiotemporal synchronization between inertial and visual data.

The FWStab dataset comprises 48 videos with synchronized sensor logs, covering diverse scenarios such as urban buildings, open skies, and sparse environments, all captured under unstable conditions. Additionally, it provides fine-grained categorization of jitter patterns induced by the flapping-wing drone’s motion modes, including high-frequency vibrations, roll motions, and pitch variations. This detailed categorization enables the network to better learn and adapt to heterogeneous jitter characteristics across motion modes, enhancing stabilization robustness in complex real-world flight scenarios.

Since datasets such as NUS [1], DeepStab [2], and MotionStab [3] do not contain gyroscope data, the experiments conduct quantitative evaluation and visual analysis of the proposed algorithm on the DeepFVS [5] and self-constructed FWStab datasets.

### 4.2. Evaluation Metrics and Experimental Setup

#### 4.2.1. Evaluation Metrics

To compare the performance of the proposed method with prior approaches, three widely used metrics are employed: cropping ratio, distortion, and stability score. A brief description of these metrics is provided below.

(1) **Crop Ratio**

The crop ratio quantifies the effective field of view retained in a video after removing black borders induced during stabilization. It is typically computed by extracting the scaling factor from the affine component of the estimated homography matrix $H_{t}$ between corresponding frames of the input and stabilized videos. The overall crop ratio is obtained by averaging these scaling factors across all frames. A higher crop ratio indicates less aggressive cropping, higher reconstruction quality, and maximal preservation of the original scene content while achieving stabilization.

(2) **Distortion**

The Distortion Score quantifies the degree of geometric distortion introduced during video stabilization. Specifically, anisotropic scaling is estimated via the ratio of the maximum to minimum eigenvalues derived from the affine components of the homography matrix $H_{t}$. A significantly large ratio indicates noticeable stretching or warping in certain frames, which degrades overall visual quality. In practical evaluations, the worst-case ratio across all frames or its average is typically used as the Distortion Score for the entire video, thereby characterizing the extent of distortion.

(3) **Stability**

The Stability Score measures the smoothness of the stabilized video by analyzing the energy distribution of motion parameters between adjacent frames in the frequency domain. Once the homography matrix for any two frames is obtained, the translation and rotation components are separated and treated as one-dimensional time series. After performing a frequency domain transformation on these series, the ratio of low-frequency energy to total energy is calculated. A higher ratio indicates smoother motion. The final stability score for the video is usually determined as the lowest energy ratio of translation and rotation, effectively reflecting the coherence and visual quality of the overall scene.

#### 4.2.2. Experimental Setup

The proposed method is compared with traditional approaches [14], YouTube Stabilizer [13], and four recent learning-based methods [2,5,41,42]. Due to the unavailability of training code [5,42] or reliance on ground-truth data [2,41], pre-trained models are utilized for the comparisons.

Additionally, among sensor-based methods, a comparison with [43] was infeasible due to the unavailability of its code and dataset. Instead, for methods leveraging both image and inertial sensor data, the proposed approach is benchmarked against the deep learning-based method DeepFVS [5] and the commercial sensor stabilization tool Gyroflow. When evaluating the custom dataset FWStab using Gyroflow, the IMU orientation is set to yXZ, while the default XYZ orientation is used for DeepFVS as its dataset does not specify IMU alignment. The FWStab dataset was divided into 32 videos for training and 16 videos for testing. The test set was further categorized into five classes: urban buildings, open skies, sparse environments, panning, and rolling. An 8-fold cross-validation was performed on the training set. Additionally, the proposed method was evaluated using the publicly available dataset released in DeepFVS.

The computational resources employed in this study include an Ubuntu 20.04.6 LTS operating system running on a server equipped with an Intel^®^ Xeon(R) Gold 6133 CPU @ 2.50 GHz (80 cores) and four NVIDIA GeForce RTX 3090 GPUs for parallel training. The deep learning framework leverages PyTorch 1.13.1, with supporting libraries including NumPy 1.23.0 and OpenCV-Python 4.8.0.76 for data processing and visualization.

### 4.3. Flapping-Wing Scenario Comparison Experiments

(1) **Quantitative Evaluation**

To validate the effectiveness of the proposed video stabilization algorithm in addressing jitter issues in videos captured by flapping-wing aerial vehicles, this section evaluates its performance on the test set of the self-constructed Biomimetic Flapping-Wing Aerial Vehicle Video Stabilization Dataset (FWStab). Seven state-of-the-art stabilization algorithms are selected for comparative experiments, including YouTube Stabilizer [13], MeshFlow [14], StabNet [2], DIFRINT [42], DUT [41], DeepFVS [5], and Gyroflow. A systematic quantitative evaluation is conducted, with results summarized in Table 2. As shown in Table 2, the method proposed in this paper demonstrates the best comprehensive performance. On the premise of effectively controlling the degree of distortion and cropping range, this method achieves the highest stability score, indicating that while improving the video stabilization effect, it can maximize the preservation of the visual quality and perspective information of the original image. Notably, YouTube Stabilizer [8] and DIFRINT [42] generated results with nearly full-frame or minimal cropping, whereas our method demonstrates comparable crop ratio performance to Gyroflow’s dynamic scaling strategy. This further validates its exceptional balance between stabilization effectiveness and cropping control.

The experiments employ three widely used evaluation metrics—Stability Index, Distortion Rate, and Cropping Ratio—to comprehensively assess the trade-off between video quality preservation and field-of-view utilization across different stabilization methods.

The evaluation results demonstrate that the proposed method achieves the best overall performance. By effectively controlling distortion levels and cropping extents, our approach attains the highest stability score, indicating its ability to enhance video stabilization while maximally preserving the original visual quality and field-of-view information. Notably, YouTube Stabilizer [8] and DIFRINT [42] produce near-full-frame or uncropped video outputs, whereas our method’s cropping ratio is comparable to Gyroflow which employs dynamic scaling, further validating its superior balance between stability and cropping control.

**Figure 6:**
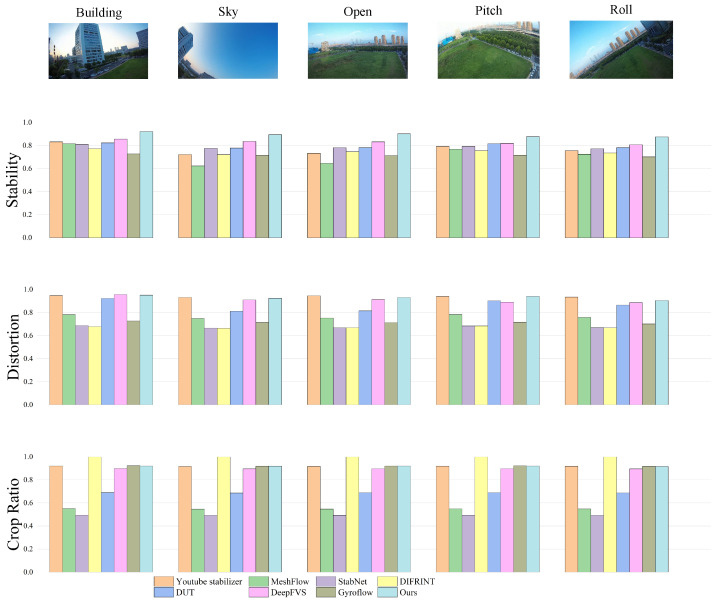
Quantitative evaluation results statistics in various scenarios on the FWStab dataset.

To more intuitively demonstrate the seven comparative algorithms and the algorithm proposed in this paper, Figure 6 shows the statistical results of the performance of each algorithm in five different scenarios (sky, high-altitude, open areas, complex texture regions, and extreme roll scenarios), which are compared in terms of stability, distortion control, and cropping control. It can be clearly observed from Figure 6 that in low-texture environments such as sky and open areas, the algorithm in this paper significantly outperforms other methods; in complex scenarios with large-scale roll, the method in this paper still maintains the optimal stability performance.

In summary, the proposed stabilization algorithm demonstrates superior stabilization performance on the self-constructed flapping-wing video dataset, FWStab. It is particularly effective in handling non-rigid jitter and complex dynamic scenarios commonly encountered by flapping-wing drones, showcasing promising application potential.

(2) **Qualitative Evaluation**

**Figure 7:**
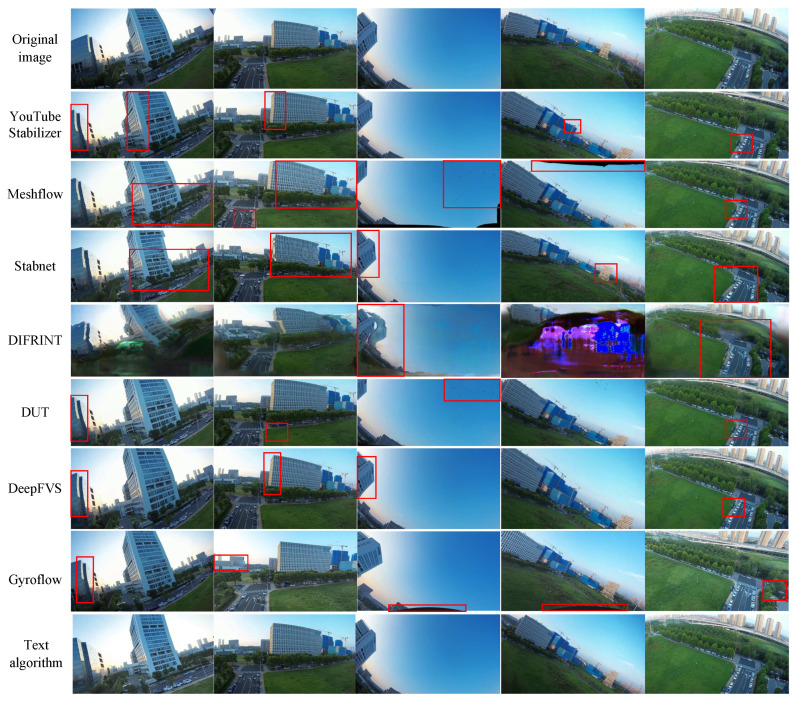
Visual comparison on FWStab dataset.

To further intuitively validate the performance of the proposed stabilization algorithm, video segments from five typical scenarios in the FWStab dataset are selected for visual comparison. The visualization results for each algorithm are shown in Figure 7. In Figure 7, five original frames from a video are intercepted and processed by seven comparative algorithms and the algorithm in this paper respectively. To further intuitively verify the performance of the image stabilization algorithm proposed in this paper, a video clip from each of the five typical scenarios in the FWStab dataset was selected for visual comparison. Other methods showed warping and distortion of varying degrees, and even large-area artifacts appeared in the roll scenario, while the method in this paper achieved excellent image stabilization results and corrected the distortion caused by the rolling shutter.

From the visual comparison, it is evident that Meshflow [10] and DUT [41], which use mesh transformations, both exhibit varying degrees of distortion in their frames; DIFRINT [42] and StabNet [22], which aim for full-frame stabilization, both show significant distortion, especially DIFRINT [42] with its iterative optimization approach, resulting in extensive distortion and artifacts in rolling scenes; YouTube Stabilizer [8] and Gyroflow do not show obvious distortion or artifacts, but their stabilization effects are mediocre; DeepFVS [37], which employs gyroscopes for stabilization, performs well in most scenarios, but struggles with distortion caused by the camera’s rolling shutter at the frame edges, which our proposed algorithm effectively corrects. Considering all factors, the video stabilization method proposed in this paper performs best in jittery videos captured by bio-inspired flapping-wing drones.

### 4.4. Comparative Experiments in Everyday Environments

(1) **Quantitative Evaluation**

To validate the generalization capability of the proposed video stabilization algorithm, this subsection conducts experiments on the public DeepFVS dataset [37] featuring everyday life scenarios. Seven state-of-the-art stabilization algorithms, namely YouTube Stabilizer [8], MeshFlow [10], StabNet [22], DIFRINT [42], DUT [41], DeepFVS [37], and Gyroflow, are selected for comparative experiments. Subsequently, a systematic quantitative evaluation is performed to assess the performance of each method.

The same three widely used evaluation metrics Stability, Distortion Rate, and Cropping Rate are employed to comprehensively assess the trade-off between video quality and field-of-view preservation across all stabilization methods. The detailed evaluation results are summarized in Table 3, which aggregates the overall performance of each method on the DeepFVS [37] test set. Table 3 shows the quantitative evaluation results on the public dataset DeepFVS. The quantitative evaluation results indicate that, compared with the DeepFVS method, the proposed algorithm improves stability by 2.46%, reduces the distortion rate by 4.69%, and decreases the cropping rate by 3.20%. Specifically, it achieves the highest scores in both stability and distortion rate, while the cropping rate score ties for the second place with Gyroflow.

The quantitative evaluation results indicate that, compared to the DeepFVS approach, the proposed method improves stability by 2.46%, reduces the distortion rate by 4.69%, and lowers the cropping rate by 3.20%. Specifically, he proposed method attains the highest scores in both stability and distortion rate, and its cropping rate score is nearly on par with that of Gyroflow. Although there remains an approximate 6.5% gap relative to full-frame video stabilization algorithms without cropping, this cropping level suffices to satisfy the demands of video stabilization algorithms in most scenarios.

(2) **Qualitative Evaluation**

**Figure 8:**
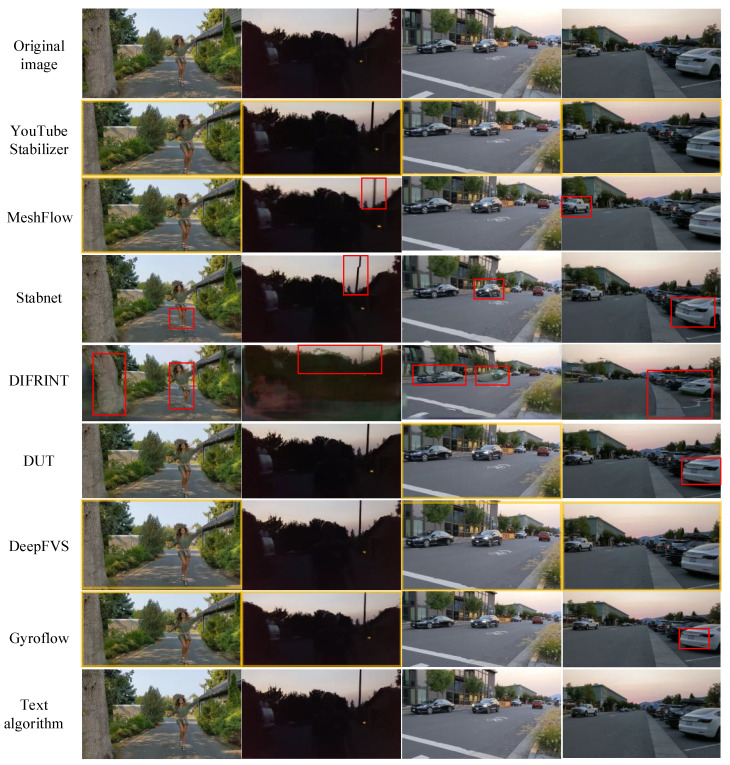
Visual comparison on DeepFVS dataset.

To further visually validate the performance of the proposed stabilization algorithm on the DeepFVS [8] dataset, frames are extracted from stabilized videos of selected clips, encompassing general jitter scenarios, foreground motion scenarios, and low-light/dark environments. The comparative visualization results of all methods are presented in Figure 8. Figure 8 shows the visual comparison results on the public dataset DeepFVS. When there is large foreground motion, MeshFlow [10] and StabNet [22] exhibit more distortions, while DeepFVS [37], Gyroflow, and the algorithm in this paper with sensor assistance show almost no distortion; although DIFRINT [42] can synthesize clear videos without any cropping, iterative optimization makes the performance of DIFRINT [42] very unstable, easily causing distortion and artifacts in the image; from the picture, the algorithm in this paper obviously has a larger field of view than DeepFVS [37] in terms of image visual range.

From the visual comparison, it is evident that when there is significant foreground motion, MeshFlow [14] and StabNet [2] exhibit more distortion. In contrast, DeepFVS [5], Gyroflow, and the proposed algorithm, which utilize sensor assistance, show almost no distortion in their outputs. Although DIFRINT [42] can synthesize clear videos without any cropping, its iterative optimization results in highly unstable performance, often leading to distortion and artifacts in the images. Visually, the proposed algorithm clearly offers a larger field of view compared to DeepFVS [5]. In terms of distortion, while the proposed algorithm shows numerical improvements over DeepFVS [5], it becomes challenging to distinguish the differences from a single image.

### 4.5. Ablation Studies

To further verify the changes in motion characteristics of flapping-wing aircraft before and after image stabilization, this paper conducted a time-domain visualization analysis of raw IMU data. The figure displays the variations in angular velocity and acceleration signals during flight, clearly showing the periodic disturbances caused by flapping vibrations. Based on this, we further carried out ablation experiments to compare and analyze the smoothness of angular velocity trajectories before and after stabilization, so as to evaluate the effectiveness of the proposed method in suppressing jitter.

**Figure 9:**
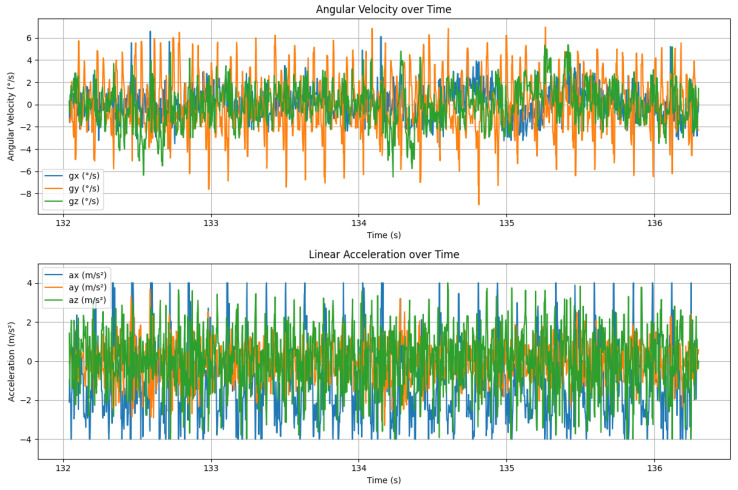
Time-domain waveforms of the gyroscope and accelerometer.

As shown in Figure 9, the original angular velocity signals (especially in roll and pitch directions) exhibit distinct periodic oscillatory waveforms, primarily derived from the high-frequency mechanical vibrations caused by the flapping mechanism. The acceleration signals also demonstrate significant periodic disturbances, most prominent in the vertical direction, reflecting the direct impact of upstroke-downstroke wing motions on the airframe’s linear vibrations.

These time-domain signals clearly reveal the significant angular momentum disturbances and linear vibrations during flight, which are the main physical sources of onboard image jitter. Thus, to verify the effectiveness of the proposed stabilization algorithm in suppressing flapping-induced vibrations, we further designed comparative ablation experiments to analyze the variations in angular velocity trajectories before and after stabilization.

Experiment 1: Validating the Effectiveness of the Foreground Removal Module. To verify the impact of the foreground removal module, we visualize the optical flow maps before and after processing by this module. As shown in Figure 5c, the module isolates foreground dynamic motion from global background motion by masking out regions affected by moving objects. The resulting optical flow retains only the background motion components. Figure 5d further demonstrates the refined optical flow after applying diffusion-based inpainting to fill in the masked regions, effectively reconstructing a globally consistent motion representation.

**Figure 10:**
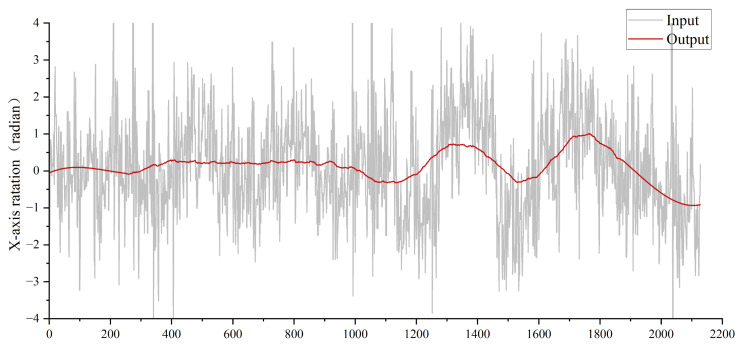
Comparison of input camera X-axis rotation trajectory and stable trajectory results.

Experiment 2: Validating the Effectiveness of LSTM. As illustrated in Figure 10, the LSTM unit integrates temporal information (e.g., motion dynamics) into the model, enabling it to generate state-specific outputs. By leveraging this temporal context, the LSTM mitigates high-frequency noise and produces more stable pose estimates. Since similar motion patterns correspond to analogous relative poses, the model can more effectively infer motion dynamics from rotational deviations rather than absolute poses. This reliance on relative poses also enhances numerical stability during training. By contrasting with methods based on absolute poses, the use of relative poses effectively highlights the true camera motion patterns in jittery videos.

**Figure 11:**
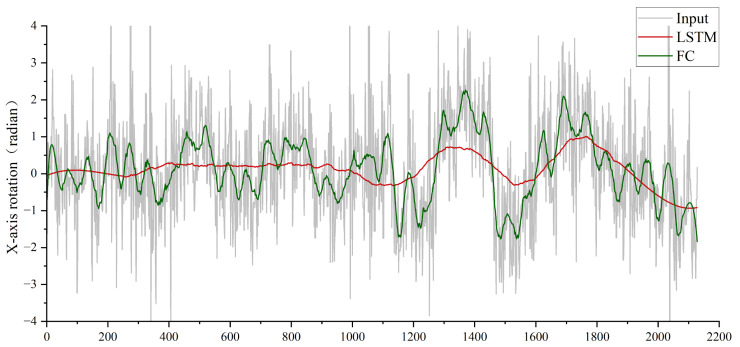
Input camera X axis rotation trajectory, FC replacement LSTM and stable trajectory results comparison.

The x-axis rotation of the camera represents vertical directional rotation on the horizontal plane, while the y-axis rotation corresponds to horizontal directional rotation (orthogonal to the x-axis). Notably, x-axis rotation is the primary source of jitter in bio-inspired flapping-wing drones due to their inherent wing-beat dynamics. To analyze camera jitter in flapping-wing drones, we focus on x-axis rotational deviations. As shown in Figure 11, replacing the LSTM module with fully connected (FC) layers introduces significantly higher jitter in the output camera poses, resulting in unstable video stabilization and degraded visual quality. This underscores the LSTM’s critical role in temporal motion smoothing and robust pose estimation for flapping-wing drone videos.

Experiment 3: Validating the Effectiveness of the Multimodal Signal Fusion Module. Similar to Experiment 2 (LSTM validation), replacing the multimodal fusion module with direct concatenation of high-level features from optical flow and IMU sensors via FC layers severely degrades performance. As shown in Figure 11, this naive concatenation prevents the LSTM from effectively predicting smooth camera poses, resulting in significantly higher jitter compared to the proposed fusion module. The multimodal fusion module explicitly models cross-modal dependencies, enabling the LSTM to leverage complementary motion cues (visual-inertial synergy) for robust trajectory estimation. This ablation study underscores the necessity of hierarchical feature interaction rather than simple concatenation for multimodal stabilization tasks.

**Figure 12:**
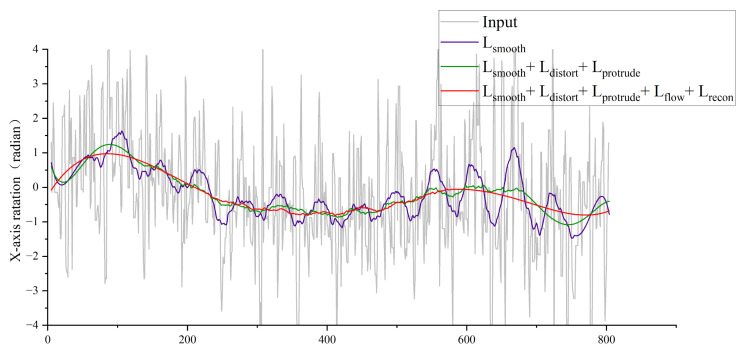
Ablation experiment.

Experiment 4: Validating the Effectiveness of the Loss Function. As illustrated in Figure 12, the multi-stage training strategy—where loss functions are incrementally incorporated during training—demonstrates significant improvements in stabilization performance. Specifically, the x-axis rotational jitter (measured by the magnitude of rotational deviations) is substantially reduced, resulting in smoother motion trajectories and progressively enhanced camera stability. This ablation study highlights the efficacy of the phased loss integration approach in refining pose estimation and mitigating high-frequency jitter artifacts.

### 4.6. Limitations of the Algorithm and Future Work

While the proposed multi-modal signal fusion video stabilization method demonstrates exceptional performance in scenarios with severe flapping-wing drone jitter, it still exhibits certain limitations in extreme motion and complex environments. First, during high-frequency nonlinear motions such as abrupt rolling or rapid pitching, the camera trajectory becomes highly erratic and irregular. The existing LSTM-based trajectory modeling struggles to fully capture such complex dynamics, often resulting in localized stabilization deficiencies or reduced compensation accuracy. Second, in adverse weather conditions like rain, snow, or fog, image quality degradation significantly impairs the performance of the optical flow estimation and foreground dynamic separation modules, forcing the system to rely more heavily on IMU data. However, when the IMU suffers from noise, drift, or synchronization errors, these issues amplify cumulative trajectory errors in stabilization, ultimately degrading video quality. Additionally, the method’s multi-stage pipeline—encompassing optical flow extraction, dynamic foreground removal, feature fusion, and trajectory smoothing prediction—introduces computational complexity and high inference latency. This complexity restricts its direct deployment in real-time applications requiring low-latency processing. Although the cross-attention mechanism enhances the fusion capability of optical flow and IMU features, its computational complexity is relatively high in high-resolution video or long-sequence modeling.

In view of this, future work can be carried out in the following directions: On the one hand, introduce more powerful temporal modeling architectures, such as Transformer-based sequence modeling or hybrid spatio-temporal networks, to enhance the ability to represent and compensate for high-frequency extreme motions. These advanced architectures can better capture the complex temporal dependencies and dynamics in such motions, enabling more accurate camera trajectory prediction and stabilization. On the other hand, explore adaptive modality weight adjustment mechanisms. By dynamically evaluating the image quality and IMU reliability, optimize the multimodal feature fusion strategy to enhance the robustness in adverse weather conditions. This approach can adaptively allocate weights to different modalities, ensuring that the system can make the most of reliable information sources and maintain stable performance even in challenging environments. Meanwhile, future work can adopt hierarchical attention mechanisms. By designing lightweight network architectures, end-to-end sparse motion modeling, and pruning-compression techniques, we aim to reduce inference overhead and enhance the system’s overall inference efficiency and real-time processing capabilities. This will provide support for real-time video stabilization applications in unmanned aerial vehicles, online navigation, and mobile photography devices.

## 5. Conclusions

This paper addresses the challenges of intense jitter and complex perturbations faced by bio-inspired flapping-wing drones during real-world flight by proposing a deep learning-based video stabilization method that leverages multimodal signal fusion. By integrating optical flow features from images with IMU sensor data, and introducing a cross-attention mechanism to achieve precise camera pose estimation, the method utilizes an LSTM network to model temporal trajectories and ultimately generates stabilized output videos through grid-based transformations. To verify the performance of the algorithm, this paper constructs a video stabilization dataset for flapping-wing platforms, FWStab, which covers various real and complex flight scenarios and fills the gap in existing datasets in this direction. Experimental results demonstrate that the proposed method outperforms state-of-the-art approaches in both flapping-wing drone scenarios and daily-life environments, achieving superior performance across metrics such as stability, distortion control, and cropping ratio. The method exhibits strong robustness and generalization capability. For jittery videos of flapping-wing aircraft, on the self-built FWStab dataset, the stability is improved by 5.92% and the distortion rate is increased by 2.40% compared with the current state-of-the-art methods. For jittery videos in general scenes, on the public DeepFVS dataset, the stability is enhanced by 2.46%, the distortion rate is increased by 4.69%, and the cropping rate is reduced by 3.20%. Future research may further extend the method’s adaptability to additional aerial platforms and incorporate additional sensor data to enhance stabilization effectiveness.

## Figures and Tables

**Table 1:** Avatar Pro Camera parameters.

Avatar Pro Camera	Parameters
Resolution	1080 p/60 fps; 720 p/100 fps; 720 p/60 fps
Aspect Ratio	16:9/4:3
Field of View	$160^{\circ }$
Aperture	F1.6
Shutter	Rolling Shutter
Weight	9.5 g
Dimensions	19 mm*19 mm*24 mm

**Table 2:** Quantitative evaluation results on the FWStab dataset.

Algorithm	FWStab Dataset		
	Stability ↑	Distortion ↑	Crop Ratio ↑
Youtube Stabilizer [8]	0.802	0.940	0.918
MeshFlow [10]	0.810	0.779	0.548
StabNet [22]	0.790	0.672	0.493
DIFRINT [42]	0.764	0.675	1.000
DUT [41]	0.804	0.891	0.688
DeepFVS [37]	0.844	0.913	0.896
Gyroflow	0.718	0.907	0.920
Proposed Algorithm	0.894	0.935	0.918

**Table 3:** Quantitative evaluation results on the DeepFVS dataset.

Algorithm	FWStab Dataset		
	Stability ↑	Distortion ↑	Crop Ratio ↑
Youtube Stabilizer [8]	0.834	0.978	0.977
MeshFlow [10]	0.831	0.768	0.557
StabNet [22]	0.836	0.850	0.753
DIFRINT [42]	0.781	0.875	1.000
DUT [41]	0.813	0.923	0.696
DeepFVS [37]	0.853	0.937	0.906
Gyroflow	0.726	0.912	0.936
Proposed Algorithm	0.874	0.981	0.935

## Data Availability

Raw/processed data are available upon reasonable request addressed to the corresponding authors.
